# Supplementary material for: Amaranth Seeds and Sprouts as Functional Ingredients for the Development of Dietary Fiber, Betalains, and Polyphenol-Enriched Minced Tilapia Meat Gels
Source: Molecules. 2022 Dec 23;28(1):117. doi: 10.3390/molecules28010117 (PMC9822371; doi:10.3390/molecules28010117)
Supplement: Supplementary file 1 [file molecules-28-00117-s001.zip › molecules-2072945-supplementary.pdf]

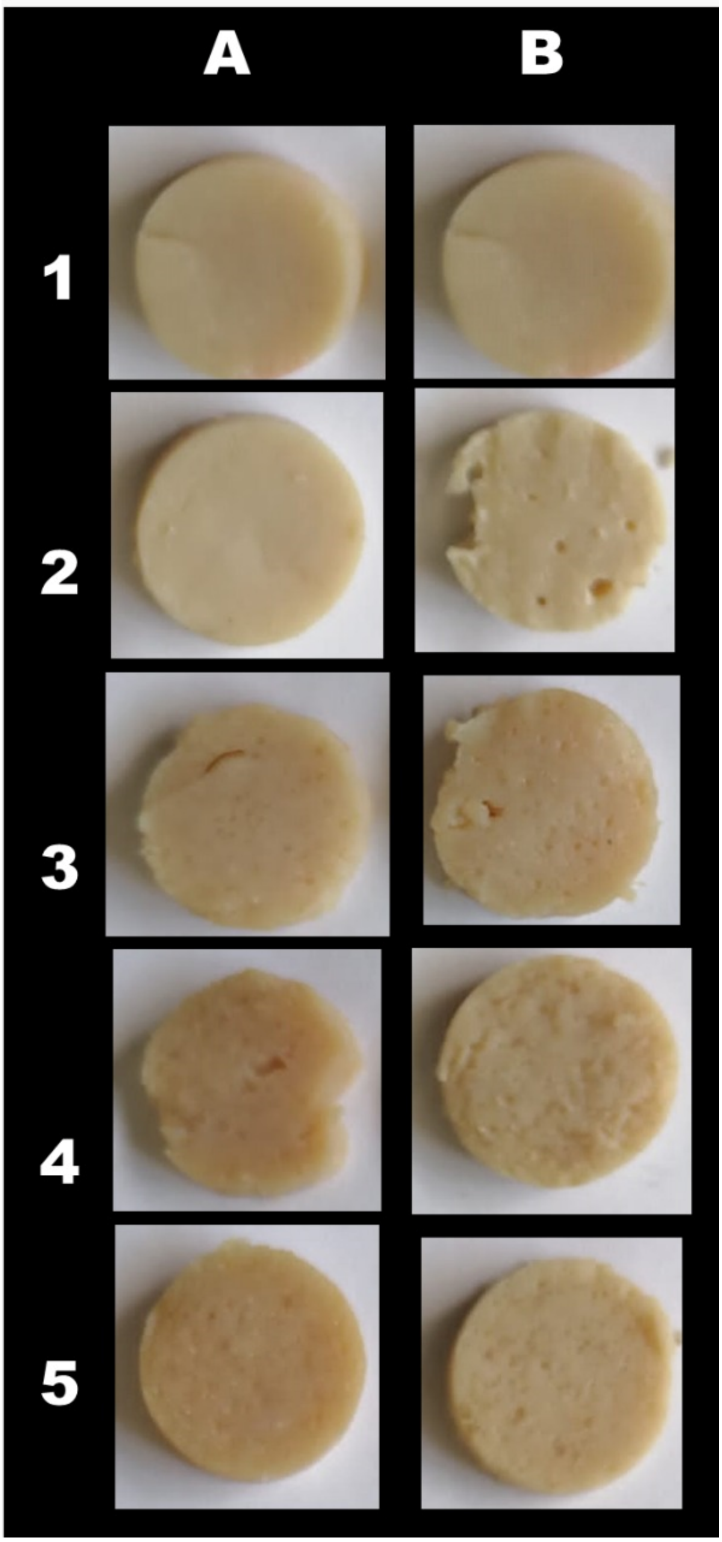

Figure S1. Representative photography of control minced tilapia meat gel (1A and 1B), minced tilapia meat gels added with 2, 4, 6, and 10% of amaranth seed flours (2A, 3A, 4A, and 5A, respectively), and minced tilapia meat gels added with 2, 4, 6, and 10% of amaranth sprout flours (2B, 3B, 4B, and 5B).

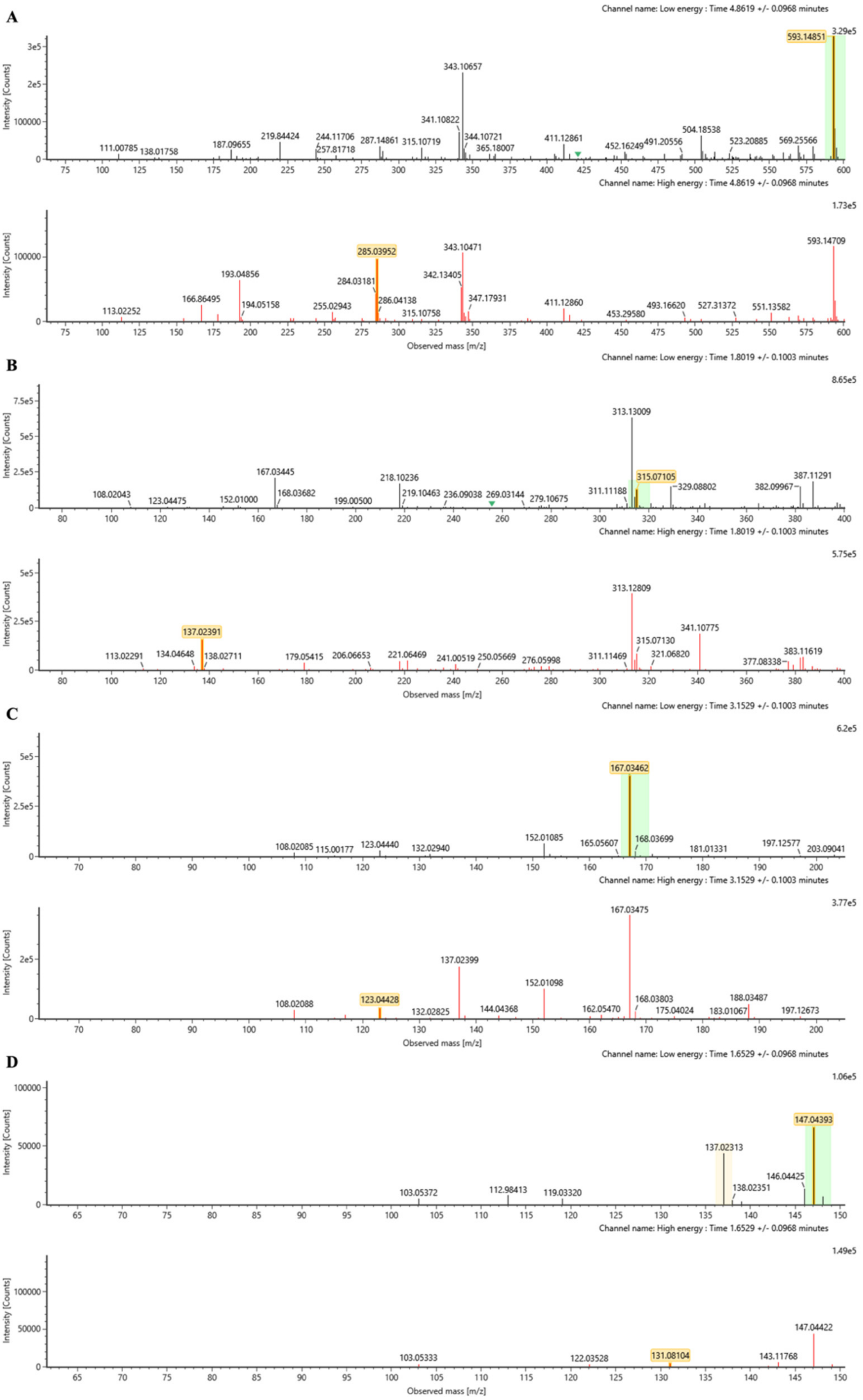

Figure S2. High resolution MS^E spectra at high (superior) and low (inferior) collision energy of the major free polyphenols identified in amaranth seeds and sprouts: kaempferol rutinoside (A), dihydroxybenzoic acid hexoside (B), vanillic acid (C), and cinnamic acid (D).

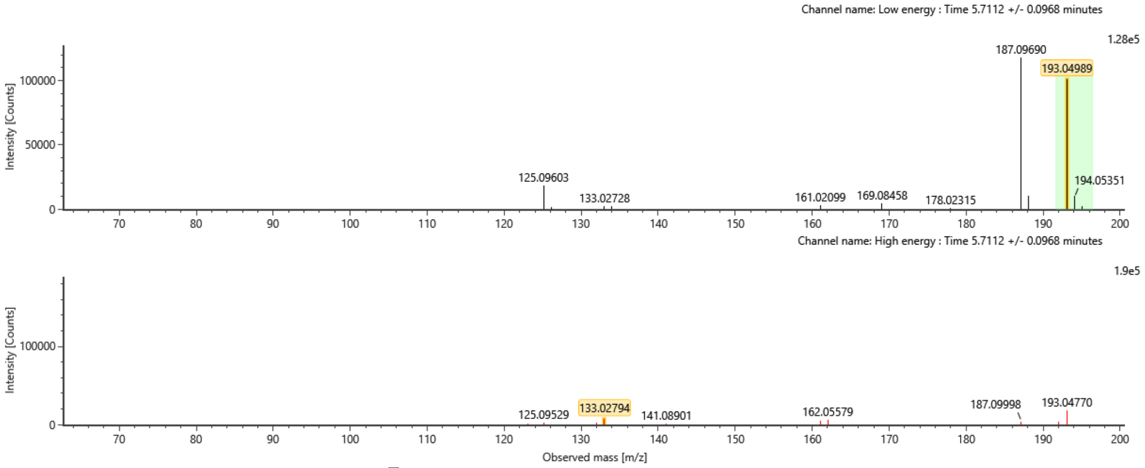

Figure S3. High resolution MS^E spectra at high (superior) and low (inferior) collision energy of ferulic acid, the major bound polyphenol identified in amaranth seeds and sprouts.

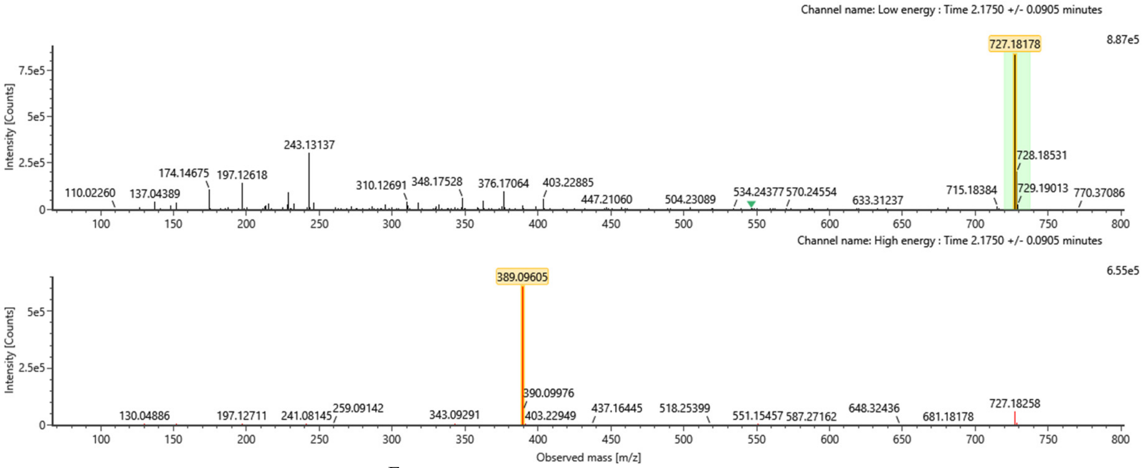

Figure S4. High resolution MS^E spectra at high (superior) and low (inferior) collision energy of betanidin β- glucuronosylglucoside (amaranthin), the major betalain identified in amaranth seeds and sprouts.

Table S1. Validation parameters of the UPLC-ESI-QTOF MS^E method.

Standard	Regression model	Model	R2 coefficient	LOD (ng/mL)	LOQ (ng/mL)	Quantified compounds
Rutin	y = 421.62 + 394.13	Linear fit	0.9981	0.02	0.06	Quercetin derivatives
Kaempferol	y =583.12 + 154.11	Linear fit	0.9992	0.01	0.02	Kampferol derivatives
Apigenin	y = 689.48 + 773.49	Linear fit	0.9983	0.02	0.08	Apigenin derivatives
Daidzein	y = 746.53 - 1021.22	Linear fit	0.9987	0.03	0.09	Daidzein derivatives
Glycitein	y = 938.49 – 874.83	Linear fit	0.9991	0.02	0.07	Glycytein derivatives
Genistein	y = 808.31 - 1149.12	Linear fit	0.9993	0.03	0.10	Genistein derivatives
4-Hydroxybenzoic acid	y = 1603.14 + 902.31	Linear fit	0.9977	0.01	0.03	Hydroxybenzoic acid and its derivatives
Vanillic acid	y = 1376.35 + 889.38	Linear fit	0.9981	0.01	0.05	Vanillic acid
Cinnamic acid	y = 4035.50 + 1603.44	Linear fit	0.9975	0.01	0.03	Cinnamic acid
Caffeic acid	y = 4489.38 – 1288.31	Linear fit	0.9992	0.01	0.02	Caffeic acid
Ferulic acid	y = 5010.94 + 1661.77	Linear fit	0.9956	0.01	0.02	Ferulic acid and its derivatives
Betanin	y = 3384.22 + 1001.39	Linear fit	0.9963	0.01	0.02	(Iso)amaranthine

Calibration curves were constructed from six different concentrations for each standard and were analyzed by triplicate. LOD: limit of detection; LOQ: limit of quantification.

Table S2. Nutritional composition of amaranth seed and sprout flours.

Component[1]	Samples	
	**Amaranth seed flour**	**Amaranth sprout flour**
Protein	17.23 ± 0.38a	15.75 ± 0.01b
Lipid	6.18 ± 0.34a	5.07 ± 0.02b
Carbohydrates[2]	67.27 ± 0.95a	70.48 ± 0.04b
Crude fiber	13.83 ± 0.75b	42.59 ± 1.27a
Ash	2.82 ± 0.13a	3.04 ± 0.13a
Moisture	6.49 ± 0.10a	5.66 ± 0.09b

Data are shown as mean ± standard deviation of three replicates. Different letters indicate significant ($p<0.05$) differences between samples by Student's t test. Data are expressed as [1] % on db. [2] Calculated by difference. db: dry basis.

Table S3. Free and bound polyphenol and betalain content of amaranth seed and sprout flours.

Component[1]	Samples	
	**Amaranth seed flour**	**Amaranth sprout flour**
Free polyphenols[1]	106.17 ± 3.20b	304.70 ± 8.17a
Free flavonoids[2]	107.66 ± 6.83b	203.08 ± 6.32a
Free proantocyanidins[3]	3.75 ± 0.30a	4.58 ± 0.19a
Bound polyphenols[1]	141.33 ± 5.97a	122.38 ± 4.52a
Betacyanins[4]	6.31 ± 0.22b	14.70 ± 0.81a
Betaxanthins[5]	3.87 ± 0.04b	5.03 ± 0.01a
Betalamic acid[6]	6.59 ± 0.17a	6.84 ± 0.17a

Data are shown as mean ± standard deviation of three replicates. Different letters indicate significant ($p<0.05$) differences between samples by Student's t test. Data are expressed as [1]mg of gallic acid equivalents/100 g db; [2]mg of rutine equivalents/100 g db; [3]mg of proantocyanidins equivalents/100 g db; [4]mg of betacyanin equivalents/100 g db; [5]mg of betaxanthin equivalents/100 g db; [6]mg of betalamic acid/100 g db. Db: dry basis.

Table S4. Free and bound polyphenol and betalain profile of amaranth seed and sprout flours.

Compound	Rt (min)	Molecular formula	Expected mass (Da)	Observed mass (Da)	Error (ppm)	Adducts	Fragments	Concentration[1] Amarnath seed flour	Concentration[1] Amaranth sprout flour
**Free polyphenols**									
Flavonols									
Quercetin rutinoside (rutin)*	3.96	C27H30O16	610.1534	609.1505	7.1979	$[M-H]^-$	301.06609,151.00078	2.33 ± 0.08b	4.89 ± 0.09a
Kaempferol rutinoside	4.86	C27H30O15	594.1585	593.1485	-4.5216	$[M-H]^-$	285.03952	5.89 ± 0.03b	10.58 ± 0.46a
Hydroxybenzoic acids									
Hydroxybenzoic acid hexoside	1.57	C13H16O8	300.0845	299.0751	-7.2877	$[M-H]^-$	137.02336,108.02008	2.34 ± 0.01b	3.67 ± 0.18a
Hydroxybenzoic acid*	1.64	C7H6O3	138.0317	137.0231	-9.3752	$[M-H]^-$	108.02008	ND	4.18 ± 0.08
Dihydroxybenzoic acid hexoside	1.79	C13H16O9	316.0794	315.0711	-3.1993	$[M-H]^-$	152.99288,137.02348,101.02349	8.69 ± 0.46a	5.16 ± 0.09b
Vanillic acid*	3.16	C8H8O4	168.0423	167.0344	-3.1996	$[M-H]^-$	123.04428,108.02088	19.57 ± 0.65a	11.83 ± 1.38b
Hydroxycinnamic acids									
Cinammic acid*	1.65	C9H8O2	148.0524	147.0439	-8.3188	$[M-H]^-$	131.08104, 203.05333	4.32 ± 0.37a	1.85 ± 0.13b
Ferulic acid hexoside	2.90	C16H20O9	356.1107	355.1024	-2.8468	$[M-H]^-$	193.04982,179.03315,134.03649	0.88 ± 0.01b	1.18 ± 0.04a
Caffeic acid*	3.02	C9H8O4	180.0423	179.0338	-6.6884	$[M-H]^-$	135.04407	ND	2.27 ± 0.22
Feruloylquinic acid	3.93	C17H20O9	368.1107	367.1008	-7.3119	$[M-H]^-$	193.05008,191.01920,134.03676	ND	0.79 ± 0.02
Ferulic acid*	5.71	C10H10O4	194.0579	193.0499	-3.8388	$[M-H]^-$	178.02315,133.02794	ND	1.82 ± 0.03
**Bound polyphenols**									
Hydroxycinnamic acids									
Ferulic acid*	5.71	C10H10O4	194.0579	193.0505	-0.5000	$[M-H]^-$	178.02315,133.02794	ND	10.24 ± 0.01
**Betalains**									
Betacyanins									
Betanidin β-glucuronosylhexoside (amaranthin)	2.19	C30H34N2O19	726.1756	727.1818	-1.4827	$[M+H]^+$	551.15457,389.09605	ND	2.40 ± 0.05
Isobetanidin β-glucuronsylhexoside (isoamaranthin)	2.57	C30H34N2O19	726.1756	727.1829	0.0657	$[M+H]^+$	551.15457,389.09688	ND	0.33 ± 0.01

Data are shown as mean ± standard deviation of three replicates. Results are expressed as mg/100 g db. Data are expressed as [1]µmol Trolox equivalents/100 g db. Different letters indicate significant ($p<0.05$) differences between samples by Student's t test. *Identification confirmed with commercial standards. Db: dry basis.

Table S5. Total antioxidant capacity of amaranth seed and sprout flours.

**Antioxidant capacity**	**Samples**	
	**Amaranth seed flour**	**Amaranth sprout flour**
Q-ABTS assay	9.69 ± 0.72b	119.34 ± 3.84a
Q-DPPH assay	36.12 ± 2.41b	56.60 ± 2.91a

Data are shown as mean ± standard deviation of three replicates. Different letters indicate significant ($p<0.05$) differences between samples. Q: QUENCHER; ABTS: 2,2′-azino-bis(3-ethylbenzo-thiazoline-6-sulfonic acid); DPPH: 2,2-diphenyl-1-picrylhydrazyl; db: dry basis; ND: not detected.
